# Mast cell mediators in hereditary angioedema

**DOI:** 10.1186/s13023-026-04294-6

**Published:** 2026-03-14

**Authors:** Hanga Réka Horváth, Noémi Andrási, Eszter Nagy, Éva Imreh, Henriette Farkas

**Affiliations:** 1 Hungarian Angioedema Center of Reference and Excellence, Department of Internal Medicine and Haematology, Semmelweis University, Budapest, Hungary; 2 Doctoral School, Semmelweis University, Budapest, Hungary; 3 Pediatric Center, Tűzoltó street Department, Semmelweis University, Budapest, Hungary; 4 Department of Laboratory Medicine, Semmelweis University, Budapest, Hungary

**Keywords:** Hereditary angioedema, Mast cell, Chymase, Diamine-oxidase, Eosinophil cationic protein, Leukotriene B4, Platelet-activating factor, Tryptase

## Abstract

**Background:**

Bradykinin-mediated hereditary angioedema due to C1 inhibitor deficiency (HAE-C1INH) is often hard to differentiate from mast cell (MC) mediated diseases. MCs can be activated by numerous stimuli, leading to the release of various mediators. Recently, crosstalk between MCs and the complement system has been reported. Clinical observations report a higher prevalence of MC-mediated diseases in the HAE-C1INH population than in the general population.

**Objective:**

To investigate MC activation in HAE-C1INH.

**Methods:**

Serum samples from patients during angioedema attack (HAE-A) and during symptom-free period (HAE-SF), as well as from non-angioedema control subjects were examined. Patients and control subjects had no history of allergic disease and normal levels of both nutritional and inhalant specific IgE. Tryptase, chymase, platelet-activating factor (PAF), leukotriene B4 (LTB4), diamine oxidase (DAO), and eosinophil cationic protein (ECP) were measured.

**Results:**

Serum level of chymase was higher in both HAE-C1INH groups than in the control group (*p* = 0.020 in both comparisons), while no difference was found between the HAE-A and HAE-SF groups. DAO and ECP did not differ from the control group, but their levels increased during angioedema attack compared to the asymptomatic state (*p* = 0.018 and 0.004, respectively). No difference was found in the levels of the other mediators in any comparison. Chymase levels during the symptom-free period were significantly lower in those patients who had more than 3 HAE attack in the three months following sampling than in those who had less than that (*p* = 0.012). No other mediators showed a difference between these two groups.

**Conclusion:**

Significantly higher chymase levels in HAE-C1INH patients raise the possibility of the activation of a specific subset of MCs in HAE-C1INH patients. Elevation of DAO and ECP levels during HAE attacks indicates the involvement of bradykinin-independent pathways in edema formation during HAE attacks.

**Supplementary Information:**

The online version contains supplementary material available at 10.1186/s13023-026-04294-6.

## Background

Hereditary angioedema (HAE) is a rare disease, characterized by recurrent subcutaneous and/or submucosal swelling episodes (HAE attacks) [1]. The timing, frequency, location, and severity of the HAE attacks show a high intra- and interindividual variability; thus, the characteristics of the next HAE attack cannot be predicted [2]. The disease is most often caused by the deficiency (type1) or dysfunction (type2) of the C1 inhibitor protein (HAE-C1INH), leading to bradykinin-mediated HAE attacks [3], which do not respond to the antihistamine, glucocorticoid, and epinephrine treatment of angioedema. Therefore, in clinical practice, it is important to distinguish bradykinin-mediated HAE from mast cell-mediated angioedema [1, 4].

Mast cells (MCs) can be activated by numerous stimuli, acting on several different receptors. Receptors on the MC surface include, but are not limited to, the most widely investigated high-affinity IgE receptor, FcεRI, which is activated when IgE bound to the receptor is aggregated by a polyvalent antigen. In addition, some more recently described mechanisms include the Mas-related G protein-coupled receptor X2 (MRGPRX2), which has a wide range of endogenous and exogenous ligands, and the C3aR and C5aR complement receptors, which can be activated by the corresponding anaphylatoxins (C3a and C5a, respectively) [5, 6]. Following activation, MCs release mediators through three major pathways. The quickest one is the exocytosis of preformed mediators (histamine, heparin, and proteases (tryptase, chymase)) from the secretory granules. Activation of MCs induces de novo synthesis of lipid mediators (eicosanoids (prostaglandin D2, cys-leukotrienes, thromboxane A2), platelet-activating factor (PAF)), cytokines, chemokines, growth factors, and interferons. Finally, activated MCs also release extracellular vesicles [5].

Recently, more and more reports have been published on the crosstalk between MCs and the complement system [7]. It has been shown that MCs can secrete complement proteins C1q, C3, C4, and/or C5, depending on the subpopulation investigated. Proteases (tryptase, chymase) released from MCs are capable of activating the C3 protein, thus making local regulation possible [8]. The C3a and C5a anaphylatoxins, in turn, have their respective receptors on the surfaces of MCs, regulating the migration and activation of these cells. The C3a receptor is found on many different subpopulations of MCs, while the expression of the C5a receptor has only been documented on the surface of skin MCs [7, 9]. Moreover, several reports have been published on the connection between MC- and bradykinin mediated edema. It has been known since the 1990s that heparin proteoglycans, isolated from MCs, can activate factor XII [10]. Later, it has been shown that human MCs release heparin upon activation that leads to a drop in blood pressure and edema formation through a factor XII- and kallikrein mediated pathway [11–13]. In addition, clinical connection has been reported multiple times between HAE and MC mediated diseases [14]. In Sweden, a twofold higher prevalence of allergy, asthma, and atopic dermatitis was reported in the HAE-C1INH population compared to the whole Swedish population [15]. In Hungary, our research group found a three times higher prevalence of hypersensitivity reactions amongst HAE-C1INH patients than in the general population [16]. On the other hand, in the USA, no consistent difference was found in the prevalence of allergic rhinitis between HAE patients and the general population [17].

In the current study, our aim was to investigate MC activation in HAE-C1INH by measuring levels of MC mediators previously not investigated in plasma samples of HAE-C1INH patients.

## Methods

### Study subjects and samples

We obtained serum samples from 19 HAE-C1INH-type1 patients, both from during HAE attacks (HAE-A) and from symptom-free periods (HAE-SF), defined as at least 3 days after the previous HAE attack. The control group consisted of 20 volunteers who have never had angioedema and in whom C1 inhibitor deficiency was excluded with complement tests (normal C1 inhibitor antigenic concentration and functional activity). For both HAE-C1INH patients and control subjects, we selected non-allergic subjects based on the following criteria: (a) Subjects had no allergic disease in their medical history. (b) When tested with local standard tests (Polycheck^®^ Hungary Food 20 and Hungary Inhalation 20 kits, measured on AP BLOT ELITE instrument (DAS Italy)), no specific IgE was present for either inhalative or nutritive allergens.

All serum samples were taken by venipuncture, centrifuged at 3000 rpm for 10 min, and aliquoted and stored in our biorepository at -80 °C until measurement. No freeze-thaw cycles were performed on the samples prior to inclusion in this study. Main characteristics of patients and samples can be seen in Table 1, while Supplementary Table 1 contains detailed data on HAE-C1INH patients and HAE attacks. The study was approved by the institutional review board, and informed consent was obtained from the participants in accordance with the Declaration of Helsinki (License number: 1067-5/2018/EÜIG).

**Table 1:** Characteristics of study subjects and samples

	HAE-SF	HAE-A	Control
Number of cases	19	19	20
Median age of the patients (years; IQR)	30.16 (25.24–49.60)	30.43 (24.41–48.95)	39.18 (35.45–48.78)
Median age of the samples (years; IQR)	4.04 (1.51–5.59)	4.67 (1.93–6.46)	5.57 (5.47–7.41)
Percentage of females	57.9%	57.9%	40.0%
Other comments	More severe patients: 8 (42.1%)	HAE attack localization (number of patients): - abdominal (4) - subcutaneous (11) - subcutaneous in multiple locations (2) - upper airway (1) - unknown (1)	–

Abbreviations: HAE-A, The group consisting of samples of HAE-C1INH patients, taken during HAE attacks; HAE-SF, The group consisting of samples of HAE-C1INH patients, taken during symptom-free periods; More severe patients, HAE patients in the HAE-SF group who had > 3 HAE attacks in the 3 months following sampling

The current investigation was a preliminary study, where we did not consider comorbidities, HAE-related or other regular medications, or HAE attack characteristics (Supplementary Table 1) when selecting patients and control subjects. The definition of HAE attack samples was that there was a visible edema at the time of sampling. Of course, in the future, more detailed analyses are needed with uniformed sampling.

### Analyzed mediators

In this study, we investigated two MC-derived proteases (chymase and tryptase), two MC-derived lipid mediators (PAF, leukotriene B4 (LTB4)), diamino-oxidase (DAO) that plays a role in histamine degradation [18], and eosinophil cationic protein (ECP) that is released from activated eosinophils [19]. The MC mediators were chosen based on the following criteria: (a) The mediator is known to increase vascular permeability; (b) The mediator has not yet been investigated in HAE-C1INH, or the previous investigations provide limited data (Table 2). Serum tryptase and ECP levels were determined by fluoroenzyme immunoassay on Phadia 100 instrument (Thermo Fisher Scientific). All other mediators were measured by commercially available ELISA kits according to the manufacturers’ instructions: chymase (ABIN1503467) and PAF (ABIN1116537) from AntibodiesOnline; LTB4 (KGE006B) from R&D Systems, Inc.; and DAO (K8500) from Immundiagnostik. ELISA measurements were performed with a Multiskan RC 351 microplate reader and evaluated with Genesis software (Labsystems Inc.).

**Table 2:** What we already know: previous investigations on MC mediators in HAE-C1INH patients

Mediator group	Mediator	SFP vs. HC	DA vs. HC	DA vs. SFP
Preformed mediators				
Biogenic amines	histamine	> (25) urine ns. (21)	urine ns. (21)	-
Serine proteases	chymase	> (current data)	> (current data)	ns. (current data)
	tryptase	ns. (25) (current data)	ns. (current data)	ns. (current data)
De novo synthesized mediators				
Lipid mediators	PAF	ns. (current data)	ns. (current data)	ns. (current data)
	PGI_2_	> (23)	> (23)	-
	LTB_4_	ns. (26) (current data)	ns. (current data)	> (26) ns. (current data)
Cytokines	TNFα	ns. (26) < (22, 28, 30)	> (23)	ns. (28) > (22, 23, 30)
	IL-1	IL-1β: ns. (22)	IL-1: > (23)	IL-1: > (23) IL-1β: ns. (22)
	IL-6	ns. (22)	-	> (20) ns. (22)
	IL-8	ns. (22, 26, 30)	-	ns. (22) > (30)
	IL-10	-	-	> (20)
	IL-17	> (29)	-	> (29)
	G-CSF	ns. (29)	-	> (29)
	GM-CSF	< (20) > (29)	-	> (20, 29)
	CCL5	> (26)	-	-
Growth factors	FGF	FGFb: > (29)	-	FGFb: > (29)
	VEGF	VEGF-D: ns. (24) VEGF: > (27) ns. (22)	-	ns. (22, 24)
	TGFβ	TGFβ1,2,3: < (20)	-	TGFβ1,2: > (20) TGFβ3: < (20)
Other mediators				
	DAO	ns. (current data)	ns. (current data)	> (current data)
	ECP	ns. (current data)	ns. (current data)	> (current data)

The results of each comparison between the examined groups can be seen in each field. The “smaller-larger signs” are open in the direction of the group showing higher values

Abbreviations: DA, During attack; DAO, Diamino-oxidase; ECP, Eosinophil cationic protein; FGF, Fibroblast growth factor; G-CSF, Granulocyte colony stimulating factor; GM-CSF, Granulocyte-monocyte colony stimulating factor; HAE-C1INH, Hereditary angioedema caused by C1 inhibitor deficiency or dysfunction; HC, Healthy control; IL, Interleukin; LT, Leukotriene; ns, Not significant; MC, Mast cell; PAF, Platelet-activating factor; PG, Prostaglandin; SFP, Symptom-free patient; TGF, Transforming growth factor; TNF, Tumor necrosis factor; VEGF, Vascular endothelial growth factor; vs., Versus

### Statistical tests

We performed a nonparametric Spearman correlation test to determine if there was any correlation between mediator levels and sample parameters (age of patient, sex of patient, age of sample, group (HAE-A, HAE-SF, or control)) to exclude significant results caused by difference in sample characteristics.

Both HAE groups (HAE-A and HAE-SF) were compared with the control group using the Mann-Whitney U-test and the two HAE groups were compared using the Wilcoxon signed rank test. p values, effect size, and statistical power were calculated and plotted for all comparisons.

We were also interested in the relationship between mediator levels and HAE severity. To be able to answer this question, we divided the HAE-SF group into two subgroups based on HAE attack number in the three months following sampling (less severe: =< 3 HAE attacks in the 3 months following sampling; more severe: > 3 HAE attacks in the 3 months following sampling). These subgroups were than compared using the Mann-Whitney U-test.

Statistical significance was set at *p* = 0.05. All analyses were made by Microsoft Excel, StatSoft 13.5, and GraphPad Prism 7.0 programs.

## Results

### Correlation of mediator levels with sample parameters

**Fig. 1:**
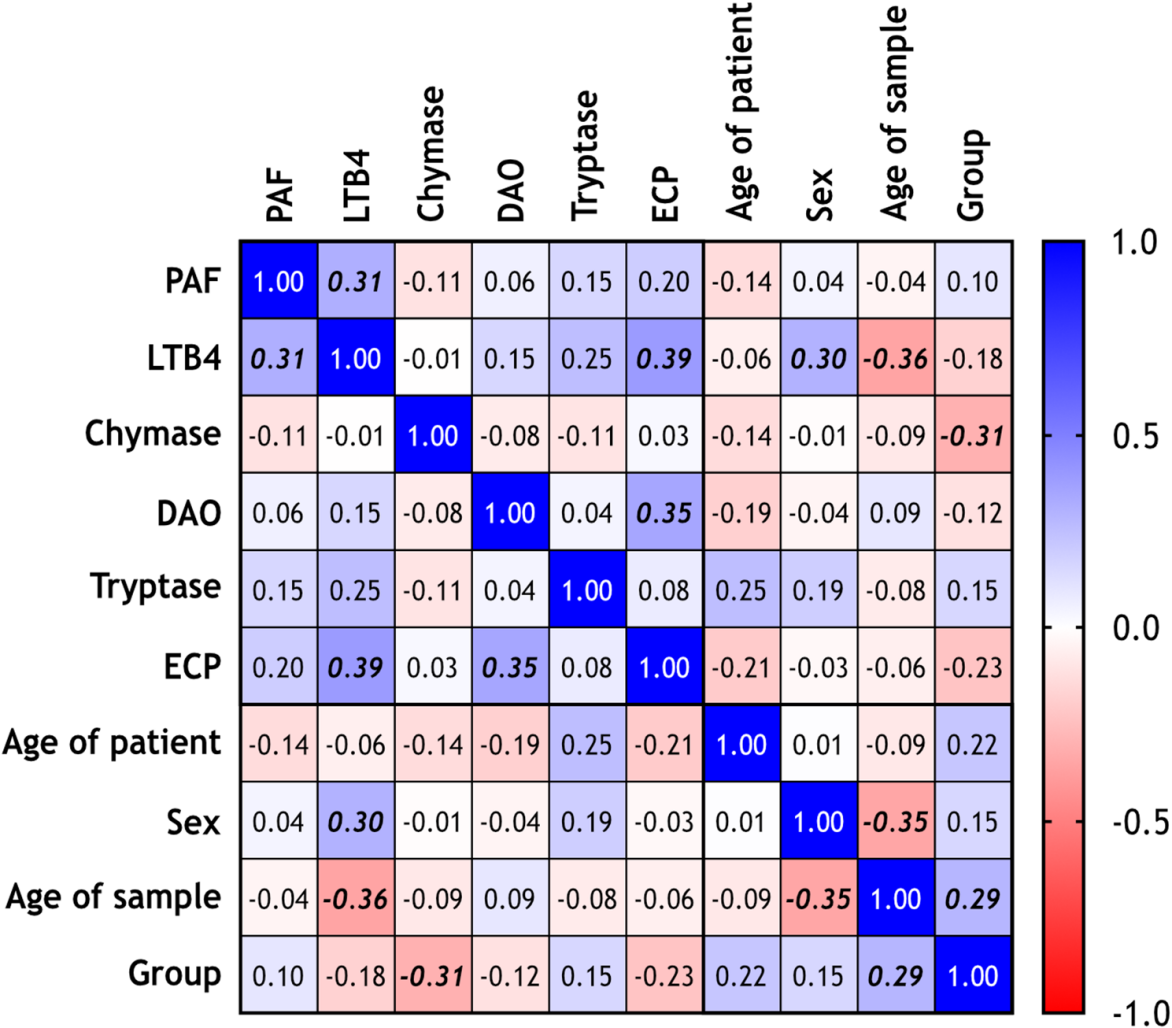
Correlation matrix of mediator levels and sample parameters. The heatmap is colored based on the magnitude of the Spearman r correlation coefficient. Bolded values represent statistically significant correlation. Abbreviations: DAO, Diamino-oxidase; ECP, Eosinophil cationic protein; LTB4, Leukotriene B4; PAF, Platelet-activating factor

Statistically significant correlation between mediator levels was found between LTB4 and PAF levels, between LTB4 and ECP levels, and between DAO and ECP levels (Spearman r 0.31, 0.39, and 0.35, respectively). Statistically significant correlation between mediator levels and sample parameters were found between LTB4 levels and the sex of the patient, LTB4 levels and the age of sample, and chymase level and group (Spearman r 0.30, -0.36, and − 0.31, respectively). The age of sample showed a statistically significant correlation with the sex of the patients and with the group (Spearman r -0.35 and 0.29, respectively) (Fig. 1).

### Levels of MC mediators

Median levels and interquartile range of the investigated mediators in each group can be seen in Table 3.

**Table 3:** Levels of the measured mediators in the three groups (median (IQR))

	PAF (pg/ml)	LTB4 (pg/ml)	Chymase (ng/ml)	Tryptase (µg/l)	DAO (U/ml)	ECP (µg/l)
HAE-SF	788.9 (315.0–1337.9)	292.9 (139.5–373.3)	26.8 (24.0–30.2)	5.8 (4.5–7.4)	10.1 (5.1–16.8)	19.8 (12.0–33.5)
HAE-A	680.9 (443.5–1769.8)	340.9 (185.5–668.4)	26.7 (23.2–31.7)	6.1 (4.9–7.7)	14.2 (8.9–34.0)	26.7 (18.6–37.7)
Control	1013.8 (601.7–1981.0)	244.0 (196.9–349.3)	21.3 (17.0–27.4)	6.9 (5.5–9.2)	13.9 (7.8–16.9)	21.6 (15.2–28.8)

Abbreviations: DAO, Diamino-oxidase; ECP, Eosinophil cationic protein; HAE-A, The group consisting of samples of HAE-C1INH patients, taken during HAE attacks; HAE-SF, The group consisting of samples of HAE-C1INH patients, taken during symptom-free periods; LTB4, Leukotriene B4; PAF, Platelet-activating factor

**Fig. 2:**
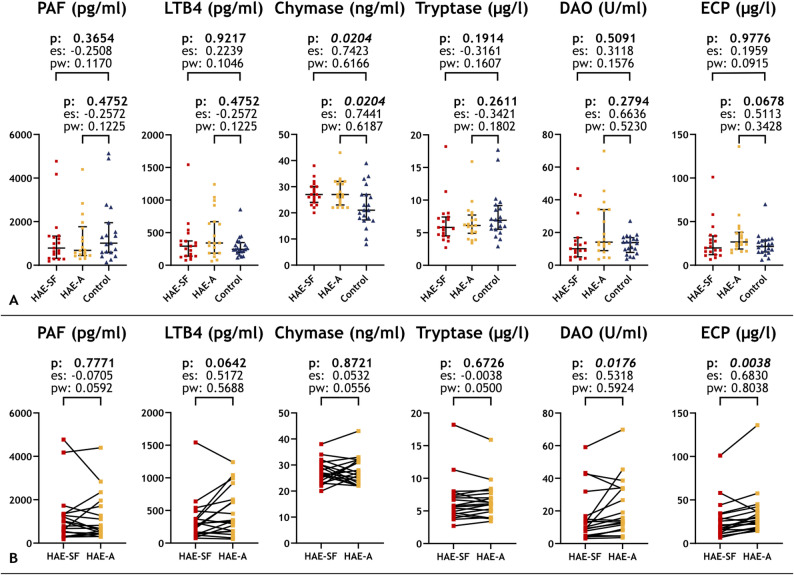
Mediator levels in all groups. Comparison of HAE-SF and HAE-A groups with the control group (**A**) and comparison of the two HAE groups (**B**). p values, effect size, and statistical power of the Mann-Whitney U-test (**A**) and the Wilcoxon signed rank test (**B**) are shown. Abbreviations: DAO, Diamino-oxidase; ECP, Eosinophil cationic protein; es: effect size; HAE-A, The group consisting of samples of HAE-C1INH patients, taken during HAE attacks; HAE-SF, The group consisting of samples of HAE-C1INH patients, taken during symptom-free periods; LTB4, Leukotriene B4; ns., Not significant; PAF, Platelet-activating factor; pw: power

Chymase levels were significantly higher in both HAE-C1INH groups when compared to controls (*p* = 0.020 for both comparisons), while in the HAE-A and HAE-SF comparison, we could not find any significant difference. DAO and ECP levels did not differ significantly from the control group, however, their levels were significantly higher during HAE attacks than in symptom-free periods (*p* = 0.018 and 0.004, respectively). Effect size and statistical power were both above 0.5 for all these comparisons. None of the three other mediators (PAF, LTB4, tryptase) showed a difference between any groups. Effect size and statistical power above 0.5 without a statistically significant difference characterized the comparison of LTB4 levels between the HAE-A and HAE-SF groups, as well as the comparison of DAO levels between the HAE-A and control groups (Fig. 2).

### Relationship of mediator levels with disease severity

**Fig. 3:**
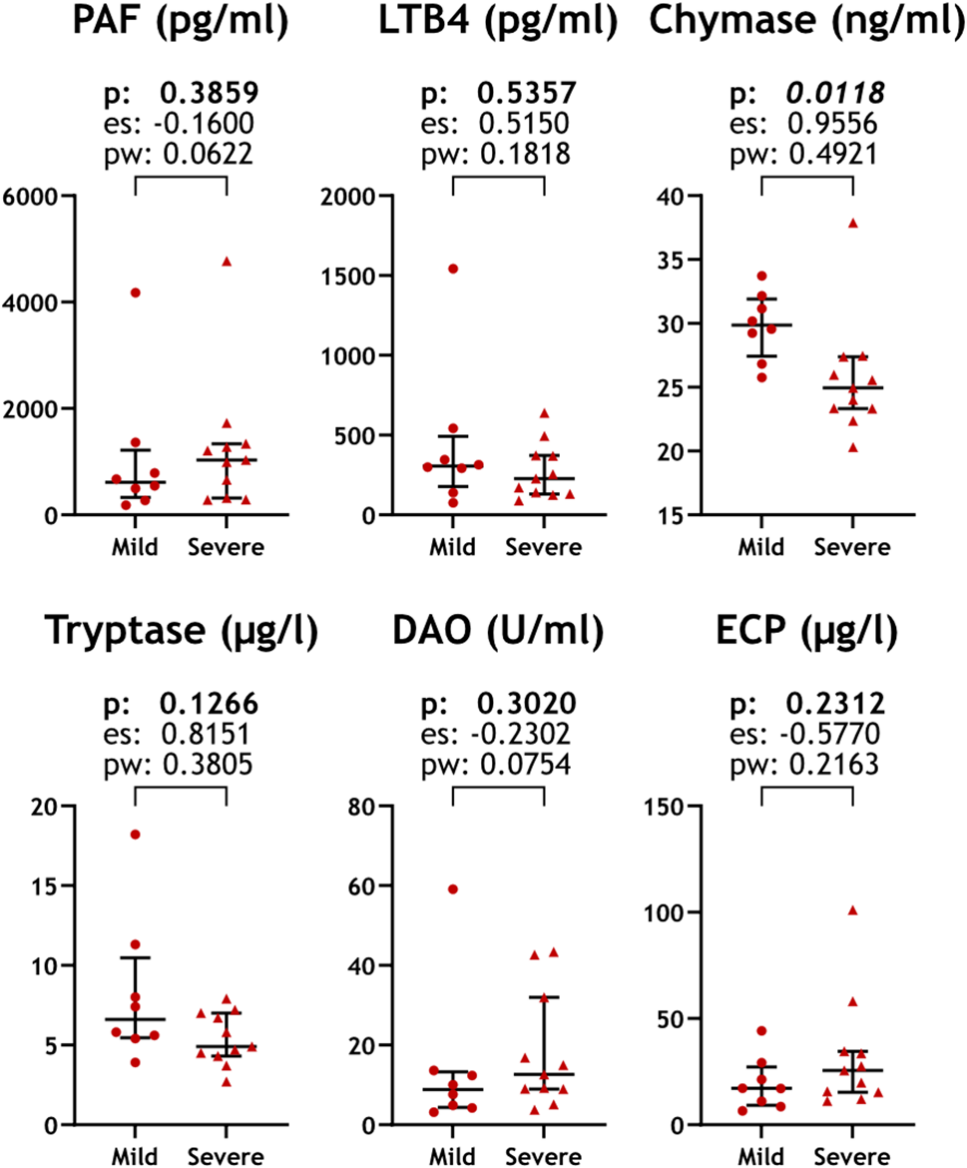
Relationship of mediator levels with disease severity. Mediator levels plotted against disease severity in HAE patients in symptom-free periods. p values, effect size, and statistical power of the Mann-Whitney U-test are shown. Abbreviations: DAO, Diamino-oxidase; ECP, Eosinophil cationic protein; es: effect size; LTB4, Leukotriene B4; ns., Not significant; PAF, Platelet-activating factor; pw: power

When comparing disease severity and mediator levels in the HAE-SF group, we found significantly lower chymase levels in the subgroup that had more severe disease than in patients with less severe disease (*p* = 0.012). Effect size was 0.9556 and statistical power 0.4921 for this analysis. No other mediators showed a difference between the two severity subgroups. A relatively high effect size (0.8151) and a statistical power of 0.3805 without statistical significance characterized the comparison of tryptase levels in the two severity subgroups (Fig. 3).

## Discussion

In our current study, we investigated four MC mediators, the histamine-degrading enzyme DAO, and the eosinophil activation marker ECP in non-allergic HAE-C1INH patients and control subjects. We observed significantly higher chymase levels in HAE-C1INH patients than in control subjects, both in symptom-free periods and during HAE attacks. DAO and ECP levels were significantly higher during HAE attacks than in the symptom-free periods, and no significant difference was observed in the tryptase, PAF, and LTB4 levels. Disease severity in the three months following sampling negatively correlated with chymase levels in the symptom-free periods.

Several MC-derived mediators have previously been investigated in HAE-C1INH patients. The results of these investigations, completed with the results of our current investigation, can be seen in Table 2 [20–30]. For most mediators, however, results have not been reported for all three comparisons (HAE-SF with Control, HAE-A with Control, and HAE-A with HAE-SF). Even if they were, it was never in one article. Moreover, in some articles, HAE attack samples were not taken from the same patients as the symptom-free samples. In our study, we investigated symptom-free and HAE attack samples of the same patients and provided results of all three comparisons.

Statistically significant correlation was found between some mediator pairs, and between some mediators and sample parameters. However, the absolute value of the correlation coefficient was lower than 0.4 in all comparisons, making the biological relevance of these correlations questionable. These results enabled us to attribute observed differences to difference between the groups and not to the potential variation in sample characteristics between groups.

Chymase is a serine protease highly abundant in mast cells, but it can also be found in cardiomyocytes or fibroblasts [31, 32]. The most well-known physiological function of the enzyme is the cleavage of angiotensin I to the active form, angiotensin II. In addition, it plays a role in the connective tissue homeostasis by facilitating tissue turnover [33]. Moreover, chymase regulates coagulation by the cleavage of thrombin and fibrinogen and by regulating plasmin activity [33, 34] and plays a role in leucocyte recruitment, partially through degrading tight junction proteins [34]. A crosstalk between chymase activity and the kinin-kallikrein system has been described. Increased bradykinin levels have been shown to correlate with increased activity of chymase in the myocardium of angiotensin converting enzyme-treated patients [35]. In vitro, chymase is able to directly release bradykinin from high molecular weight kininogen [36]. It is also known that chymase content of MCs varies greatly with tissue distribution [37]. In view of these facts, we concluded that the elevated chymase levels found in our HAE-C1INH patients might be caused by the activation of special mast cell subpopulations. During the symptom-free periods, chymase levels negatively correlated with severity of the disease in the 3 months following sampling. This result seems to contradict expectations that higher chymase levels lead to production of more bradykinin. However, it is known that a higher amount of chymase is needed for thrombin cleavage than for the cleavage of adhesion molecules [33], suggesting that lower chymase levels might lead to edema formation through an increase in thrombin levels. Nevertheless, the role of chymase in HAE-C1INH warrants further investigation.

DAO, one of the major histamine-degrading enzymes, is produced in the intestine, the kidney, and the placenta [38]. In susceptible individuals, its decreased function leads to the accumulation of histamine, provoking the symptoms of histamine intolerance [39]. It has also been shown that plasma levels of DAO are elevated during anaphylaxis in mastocytosis patients. This elevation might be caused by heparin liberating DAO from the intestinal cells [40]. In light of these data, we hypothesize that in our HAE-C1INH patients, elevated DAO levels during HAE attack might reflect either (a) elevated histamine levels through an increased need for histamine degradation; (b) damage of the intestinal mucosa, leading to DAO release; or (c) mast cell activation as described by Boehm et al. [40].

ECP, released from eosinophil granulocytes upon activation by various inflammatory stimuli, has a cytotoxic role [41, 42]. Its role has been best described in the context of airway inflammation, either in bronchial asthma or in eosinophilic chronic rhinosinusitis [43, 44]. Elevated ECP levels observed in our study during HAE attacks might indicate eosinophil activation. Together, the elevation of DAO and ECP during HAE attacks suggests that other pathways, apart from the bradykinin-forming kinin-kallikrein system, may also play a role in edema formation.

LTB4 is an arachidonic acid metabolite, synthesized de novo upon mast cell activation. It is a well-known inflammatory mediator that increases vascular permeability [45]. In a previous study focusing on neutrophil granulocytes, our group has investigated LTB4 levels in patient samples different from the ones used for the current study [26]. In both cases we found that LTB4 levels did not differ between HAE-C1INH patients during symptom-free periods and control subjects. However, in the previous study, a significant increase in LTB4 levels was observed during HAE attacks, while in the current study no such difference could be observed. This discrepancy might be attributed to a small sample size in both studies and warrants further investigation.

PAF is a lipid mediator responsible for platelet activation, the induction of proinflammatory processes, and an increase in vascular permeability [6, 13]. To the best of our knowledge, ours is the first study to investigate PAF levels in HAE-C1INH patients, and it seems rightly so, as we did not observe any differences in any comparison.

Finally, tryptase, like chymase, is a serine protease abundant in MCs. Tryptase is the major protease component of MC granules and is considered the biomarker of MC activation. Its physiological role includes local activation of the immune response, with leukocyte recruitment and vasodilation [6, 13]. Tryptase levels higher than 20 ng/ml represent a minor criterion in the diagnosis of mastocytosis [46]. In our current study we found no difference in tryptase levels between any groups, indicating that no systemic mast cell activation occurs in HAE-C1INH or during HAE attacks.

As with every research, our study also has its limitations. Relatively small sample size limits statistical power, while the prolonged duration of sample storage before analysis could serve as a confounding factor. The localization and severity of HAE attacks differed between patients which might also be a potential confounder as the relative localization of the swelling and the sampling site, as well as the severity of the HAE attack, might influence mediator levels [47]. Unfortunately, our sample size did not permit further analysis based on HAE attack localization. In the current study, only certain MC mediators could be investigated that do not fully capture activation of MCs. Finally, non-HAE angioedema controls are missing which limits our ability to conclude whether our findings are HAE-specific or reflect general angioedema physiology.

## Conclusions

To conclude, our research showed significantly higher chymase levels in HAE-C1INH patients than in non-angioedema controls, with no difference in tryptase levels. This finding might indicate the (partial) activation of specific mast cell subpopulations in HAE-C1INH patients. We have also demonstrated elevation of DAO and ECP levels during HAE attacks, indicating the involvement of bradykinin-independent pathways in edema formation during HAE attacks.

Our findings suggest the possibility of a connection between bradykinin- and mast cell mediated edema on a molecular level. However, further studies with more samples, uniformed sampling, investigating other MC mediators, and possibly allergic HAE patients or non-HAE angioedema patients as well, are needed to confirm these findings.

## Supplementary Information

Below is the link to the electronic supplementary material.

Supplementary Material 1

## Data Availability

The datasets used and/or analysed during the current study are available from the corresponding author on reasonable request.

## Abbreviations

DA: During attack
DAO: Diamine-oxidase
ECP: Eosinophil cationic protein
FGF: Fibroblast growth factor
G-CSF: Granulocyte colony stimulating factor
GM-CSF: Granulocyte-monocyte colony stimulating factor
HAE: Hereditary angioedema
HAE-A: The group consisting of samples of HAE-C1INH patients, taken during HAE attacks
HAE attack: Swelling episode in hereditary angioedema
HAE-C1INH: Hereditary angioedema caused by C1 inhibitor deficiency or dysfunction
HAE-SF: The group consisting of samples of HAE-C1INH patients, taken during symptom-free periods
HC: Healthy control
IL: Interleukin
LT: Leukotriene
LTB4: Leukotriene B4
MC: Mast cell
MRGPRX2: Mas-related G protein-coupled receptor X2
ns: Not significant
PAF: Platelet-activating factor
PG: Prostaglandin
SFP: Symptom-free patient
TGF: Transforming growth factor
TNF: Tumor necrosis factor
VEGF: Vascular endothelial growth factor
vs.: Versus
